# The DR score in RETeval™ electroretinogram system facilitates expeditious and uncomplicated early detection and assessment of diabetic polyneuropathy in clinical practice

**DOI:** 10.1371/journal.pone.0336117

**Published:** 2025-11-13

**Authors:** Yukako Sugiura-Roth, Tatsuhito Himeno, Emi Asano-Hayami, Yuriko Asada-Yamada, Miyuka Kawai, Yuka Shibata, Tomohide Hayami, Mikio Motegi, Makoto Kato, Hiromi Nakai-Shimoda, Emiri Yura-Miura, Yoshiaki Morishita, Masaki Kondo, Shin Tsunekawa, Jiro Nakamura, Hideki Kamiya

**Affiliations:** 1 Division of Diabetes, Department of Internal Medicine, Aichi Medical University School of Medicine, Nagakute, Aich, Japan; 2 Department of Clinical Laboratory, Aichi Medical University Hospital, Nagakute, Aichi, Japan; NYU Grossman Long Island School of Medicine, UNITED STATES OF AMERICA

## Abstract

**Background:**

The principal aim of this investigation was to assess the utility of a novel DR score for the early detection of diabetic polyneuropathy (DPN). This score, currently integrated into the RETeval™ electroretinogram (ERG) system, is derived from parameters such as ERG wave characteristics, patient age, and pupillary response. Traditional nerve conduction studies (NCS), though valuable, have notable limitations, including the necessity for costly equipment and specialized personnel. Consequently, it was postulated that the DR score—initially devised for predicting diabetic retinopathy—might serve as a practical alternative for diagnosing DPN. This study sought to test the hypothesis that the DR score could offer a reliable means of both diagnosing DPN and estimating its severity.

**Methods:**

The study retrospectively analyzed 82 diabetic patients admitted to Aichi Medical University Hospital between November 2016 and January 2019. ERG was performed using the RETeval™ device, and NCS was conducted to classify DPN stages according to the Baba’s Differentiation Classification (BDC) system. Multiple regression analysis and receiver operating characteristics (ROC) analysis were employed to assess the relationship between the DR score and DPN stages.

**Results:**

Among 82 participants, 24.4% (n = 20) had no DPN (stage 0), and 75.6% (n = 62) had stage 1 or higher DPN. The DR score was significantly correlated with various clinical parameters, including nerve conduction velocities and the severity of DPN as classified by BDC stages. The regression model showed that both the DR score and age were significant predictors of DPN severity. The ROC analysis demonstrated that the DR score had a moderate ability to discriminate between no DPN and stage 1 or more of DPN, with an area under the ROC curve of 0.738.

**Conclusions:**

In conclusion, this study involving 82 patients suggests that the DR score may be a valuable tool for the early detection and staging of DPN, potentially offering a more accessible and cost-effective alternative to traditional NCS, with significant implications for improving diabetic care.

## Introduction

Diabetic polyneuropathy (DPN) is one of the early manifestations of chronic diabetic complications, appearing even in the initial stages of diabetes [1,2]. A significant portion, ranging from one-third to more than half of patients, remain unaware of the onset of DPN due to the absence of symptoms in its early stages [3,4]. By the time symptoms become apparent, the condition may have advanced to its later stages, at which point the risks of foot ulcers, gangrene, and even amputation become markedly elevated, detrimentally impacting both the life quality and survival prospects of the patient. Therefore, detecting DPN in its nascent phase is crucial for improving outcomes in diabetic individuals [1,5].

Historically, electroretinography (ERG) has served as a reliable and objective technique to detect changes in retinal physiology [6]. Due to its high sensitivity, it is capable of identifying even the most subtle or subclinical retinal abnormalities. Nevertheless, the traditional ERG procedure, which necessitates the use of a contact lens electrode to capture the retinal electrical signals, is not without its drawbacks, being both somewhat invasive and time-intensive. Fortuitously, a more recent innovation has seen the advent of a portable ERG apparatus RETeval^TM^ that utilizes skin electrodes, thus simplifying the process [7]. It has been suggested that the handheld RETeval^TM^ ERG device may serve as an invaluable tool for clinicians assessing retinal function. A study has demonstrated that this device provides results closely aligned with those obtained through traditional methods involving a photic stimulator [8].

ERG is a valuable tool for evaluating the function of the retina, which, as a neuroretina, processes visual stimuli through a complex neural network and transmits this information to the cerebral cortex. Given that ERG assesses the function of the retina in its role as part of this neural system, it can also be considered a method for evaluating broader neural system functionality. In previous investigations, we have demonstrated that ERG results exhibit a commendable correlation with nerve conduction function in patients with diabetes [9]. This study aims to further explore whether the point-of-care ERG testing device could contribute to the early detection of DPN.

The motivation for this research stems from the Toronto Consensus, which emphasized the necessity of nerve conduction studies for diagnosing DPN [5]. However, nerve conduction study (NCS) require expensive equipment and highly skilled practitioners. As a result, subjective and non-quantitative symptom assessments remain widely used in various studies. The Toronto Consensus also permits the use of three additional tests, beyond nerve conduction studies, to evidence small-fiber nerve abnormalities. These include the evaluation of corneal nerve fiber degeneration, sural nerve neuropathy, and the reduction of intraepidermal nerve fibers. Assessing corneal nerve fibers requires a corneal confocal microscope. Although this method has been validated in several studies, the instrument remains prohibitively expensive [10–12]. Similarly, while sural nerve and skin biopsies are effective in evaluating neuropathy and have been validated through numerous studies [12,13], they require specialized pathological processing and are thus challenging to implement on a broad scale, in addition to being highly invasive.

In our previous work, we reported on the utility of the DPNCheck™ device, a simplified nerve conduction testing instrument, for the early diagnosis of DPN [14]. Although our findings illustrated that DPNCheck™ is valuable for diagnosing moderate to severe stages of DPN, identifying early-stage DPN remained challenging. Given that DPN is a neurodegenerative condition, early diagnosis and treatment from the outset are essential. Nonetheless, effective tools to anticipate early-stage nerve conduction abnormalities are still lacking, underscoring the urgent need for further advancements in this field.

In light of these constraints, our investigation seeks to assess the potential of the newly incorporated DR score index within the RETeval™ system for early diagnosis of DPN. The DR score, originally devised for evaluating diabetic retinopathy (DR), employs a yet proprietary algorithm not publicly disclosed. However, the parameters utilized in the calculation of the DR score—age, the implicit time and amplitude of ERG-derived waves, and pupillary response—each hold significant relevance to the early detection of DPN. Aging is widely acknowledged as a key risk factor for DPN, while a decline in autonomic nerve function, an integral aspect of DPN, may notably influence pupillary reflexes. Furthermore, our prior findings have established a correlation between the implicit time and amplitude of ERG responses and peripheral nerve conduction [14]. These observations collectively suggest that the DR score may serve as a robust predictor for the early identification of DPN. This study aims to validate this hypothesis, leveraging the predictive power of each parameter involved in the DR score.

## Methods

### Eligibility criteria

Between November 2016 and January 2019, a cohort of 82 individuals, previously diagnosed with diabetes and admitted to Aichi Medical University Hospital for the amelioration of hyperglycemia, was retrospectively analyzed in the present inquiry. Exclusion criteria included the presence of diabetic ketoacidosis, severe infection, severe injuries, or peripheral neuropathies other than DPN. Clinical data were accessed on 31/08/2024. Although no a priori power analysis was conducted because of the retrospective design, a post hoc power estimation indicated that with this sample size, a moderate correlation (r = 0.3) could be detected with approximately 80% power at a significance level of 0.05.

The characteristics of these patients were evaluated through clinical and laboratoryassessments, including physical examinations, sociodemographic details, and an array of laboratory assessments such as serum creatinine, serum urea nitrogen, urinary albumin-to-creatinine ratio, random blood glucose, glycoalbumin, glycosylated hemoglobin, high-density lipoprotein, low-density lipoprotein, and triglycerides. The estimated glomerular filtration rate (eGFR) was computed utilizing the equations promulgated by the Japanese Society of Nephrology [15]. Ophthalmological evaluations, aimed at assessing DR, were executed by qualified ophthalmologists. DR was categorized in strict accordance with the International Clinical Classification System for Diabetic Retinopathy and Diabetic Macular Edema, which delineates DR as absent, non-proliferative, or proliferative retinopathy [16]. Ankle-brachial index (ABI) and brachial-ankle pulse wave velocity (baPWV) were measured. The protocol governing this study was sanctioned by the Ethics Committee of Aichi Medical University Hospital (Ref: 2019−133) and was duly registered with the University Hospital Medical Information Network (UMIN ID: 000021916). Given the retrospective nature of this study, explicit informed consent for research participation was not obtained. Instead, the research plan was prominently displayed on the hospital’s website, allowing patients to indicate their desire to opt out, should they wish their data not to be included. This investigation was conducted in full accordance with the tenets of the Declaration of Helsinki.

### ERG

The patients underwent ERG utilizing the RETeval™ flicker ERG testing device (LKC Technologies, Gaithersburg, MD) without the induction of mydriasis. The ERG signals were detected by a skin electrode array (Sensor Strip; LKC Technologies, Inc., Gaithersburg, MD, USA) positioned 2 mm inferior to the lower eyelid margin. The flash retinal illuminance was automatically adjusted to levels of 16, or 32 photopic troland-seconds (Td-s). The flicker stimulation was delivered at a frequency of 28.3 Hz. The fundamental component’s amplitudes and implicit times were automatically computed and displayed by the RETeval^TM^ system. Amplitudes were quantified in microvolts (µV), and implicit times were measured in milliseconds (msec). The DR score assessment protocol integrates implicit time, amplitude, age, and pupil response to generate a consolidated result that is displayed immediately upon completion of the test.

### Electrophysiological studies and definition of DPN

Electrophysiological assessments were conducted using a standard electromyogram in a temperature-controlled, electrically shielded environment. The examiners were blinded to the participants’ clinical information. The focus of the evaluation was on the tibial motor nerve and the sural sensory nerve. This focus was chosen because the Baba’s Differentiation Classification (BDC) system, which categorizes DPN into stages, specifically utilizes data from these nerves [17,18].

The measured parameters included motor nerve conduction velocities (MNCV), amplitudes of compound muscle action potentials (CMAP), sensory nerve conduction velocities (SNCV), and amplitudes of sensory nerve action potentials (SNAP). F-wave latencies for the tibial nerve were recorded following 16 supramaximal stimuli, with CMAP and SNAP amplitudes assessed by peak-to-peak measurements.

To standardize the implementation of NCS and the interpretation of results, Baba et al. proposed the BDC system, a new DPN severity classification. The BDC system classifies DPN into stages from 0 to 4, based on the data from the tibial and sural nerves. NCS data were categorized as follows: Stage 0 indicates normal findings without abnormalities; Stage 1 denotes mild neuropathy with criteria including delayed tibial MNCV (<40 m/s), delayed sural SNCV (<40 m/s), prolonged tibial minimal F-wave latency (>[12.8 + 0.22 × height (cm)] ms), or the presence of an A wave; Stage 2 signifies moderate neuropathy with a sural SNAP amplitude <5 µV; Stage 3 represents moderate-to-severe neuropathy with both a sural SNAP amplitude <5 µV and a tibial CMAP amplitude between ≥2 to <5 mV; Stage 4 denotes severe neuropathy with a sural SNAP amplitude <5 µV and a tibial CMAP amplitude <2 mV. The “presence of an A wave” criterion was excluded due to low interrater reliability. To ensure accurate assessment and to mitigate potential impacts from latent orthopedic spinal disorders and radiculopathies, the higher values of nerve conduction function were used for SNCV, SNAP, MNCV, and CMAP, while the lowest value was used for minimum F-wave latency.

### The coefficient of variation of RR intervals (CV_R-R_)

The CV_R-R_ was evaluated according to established protocols [19]. ECG recordings were obtained from participants in a supine position, under both normal and deep breathing conditions, each for a duration of one minute following a five-minute period of bed rest. The first one-minute ECG recording was taken during normal breathing, followed by a one-minute recording during deep breathing at a rate of six breaths per minute. The CV_R-R_ was then calculated using the formula: CV_R-R_ (%) = (standard deviation of RR intervals)/ (mean RR intervals) × 100.

### Statistical analysis

Data analysis was conducted employing SPSS Statistics version 28.0.1.1 for Windows (IBM SPSS, Chicago, IL, USA). Pearson’s correlation coefficient was used to assess correlations among variables. Stepwise multiple regression analysis was performed with the BDC stages of DPN as the dependent variable. The DR score served as the explanatory variable, with adjustments made for age and gender to account for potential confounding factors. The regression equation obtained from this analysis provided predicted values for DPN staging.

Subsequently, ROC analysis was employed to evaluate the diagnostic performance of these predicted values. Specifically, ROC analysis assessed the ability to diagnose DPN as Stage 1 or higher and Stage 2 or higher. The area under the ROC curve (AUROC) was used to measure diagnostic accuracy. For determining the optimal cutoff values from the ROC analysis, the minimum distance method was utilized. The optimal cutoff value is calculated by minimizing the distance between the ROC curve and the point of perfect classification (0,1) on the ROC space. Mathematically, the optimal cutoff value is determined as:


$$\text{Optimal cutoff}={\text{argmin}}_{\text{x}}\text{sqrt}[(\text{1}-{\text{sensitivity}(\text{x}))}^{\text{2}}+(\text{1}-{\text{specificity}(\text{x}))}^{\text{2}}]$$

These optimal cutoff values were then used to assess the diagnostic performance of the predictions for two purposes: diagnosing DPN as Stage 1 or higher and diagnosing DPN as Stage 2 or higher. Statistical significance was established at a p-value of <0.05.

## Results

### Clinical characteristics

The clinical characteristics of the participants are detailed in Table 1. A total of 82 patients were included in the analyses, comprising 52 males and 30 females. The study population consisted of 6 patients with type 1 diabetes, 71 with type 2 diabetes, and 5 with an unspecified type. According to the International Clinical Classification System, 28% of the patients (n = 23) were classified as having non-proliferative or more severe stages of DR. Based on the BDC, 42.5% of the patients (n = 34) were categorized as stage 1 DPN, while 28.8% (n = 23) were classified as stage 2 DPN.

**Table 1:** Demographic and clinical characteristics of the participants.

	Number	
Demographic and anthropometric characteristics		
Age (years)	82	61.7 ± 14.9
Male	82	52 (63.4)
Height (cm)	81	162.5 ± 8.2
Weight (kg)	81	67.7 ± 16.3
BMI (kg/m2)	81	25.6 ± 5.3
Type of diabetes (Type 1/Type 2/Others)	77	6/71 (7.8/92.2)
Diabetes duration (years)	81	10.1 ± 10.5
Stage of Diabetic retinopathy (NDR/NPDR/PDR/TDR)	82	59/11/3/9 (72.0/13.4/3.7/11.0)
Stage of diabetic nephropathy (1/2/3/4/5)	82	50/20/5/6/1 (61.0/24.4/6.1/7.3/1.2)
Stage of diabetic polyneuropathy (0/1/2/3/4)	80	20/34/23/0/3 (25.0/42.5/28.8/0.0/3.8)
Biochemical tests		
Urea nitrogen (mg/dl)	82	15.9 ± 8.2
Creatinine (mg/dl)	82	1.0 ± 1.3
eGFR (mL/min/1.73 m2)	82	75.8 ± 28.5
uACR (mg/g)	82	214.8 ± 613.9
Total cholesterol (mg/dl)	80	185.3 ± 46.6
HDL-C (mg/dl)	81	44.2 ± 13.8
LDL-C (mg/dl)	80	107.2 ± 32.7
Triglyceride (mg/dl)	81	174.1 ± 156.2
Uric acid (mg/dl)	81	5.3 ± 1.7
AST (U/L)	82	34.4 ± 39.6
ALT (U/L)	82	32.5 ± 25.2
Random blood glucose (mg/dl)	81	195.3 ± 99.4
GHb (%)	82	10.6 ± 2.5
GHb (mmol/mol)	73	28.8 ± 10.2
Urine C-Peptide (µg/day)	80	62.3 ± 57.0
Neurovascular assessment		
MNCV, tibial nerve (m/s)	80	42.3 ± 4.4
Amp. of CMAP, tibial nerve (mV)	80	15.9 ± 7.6
F-min, tibial nerve (ms)	75	51.0 ± 4.4
SNCV, sural nerve (m/s)	77	46.2 ± 7.9
Amp. of SNAP, sural nerve (µV)	77	9.2 ± 7.1
SBP (mmHg)	82	127.6 ± 19.1
DBP (mmHg)	82	74.7 ± 12.7
CVR-R, resting (%)	76	2.6 ± 1.4
CVR-R, deep breathing (%)	73	4.3 ± 2.4
baPWV (cm/s)	77	1614.7 ± 364.6
Ankle-brachial index	77	1.1 ± .1
DR score, ERG	82	19.9 ± 5.1
ERG implicit time, 32 Td-s (ms)	82	29.9 ± 3.8
ERG amplitudes, 32 Td-s (µV)	82	20.8 ± 9.0
ERG implicit time, 16 Td-s (ms)	82	31.0 ± 3.7
ERG amplitudes, 16 Td-s (µV)	82	17.7 ± 7.9

Note: Categorical variables are given as number (percentage) while continuous variables are reported as mean ± standard deviation. NDR: no diabetic retinopathy, NPDR: non-proliferative diabetic retinopathy, PDR: proliferative diabetic retinopathy, TDR: treated diabetic retinopathy, eGFR: estimated glomerular filtration rate, uACR: urine albumin-to-creatinine ratio, HDL-C: High-density lipoprotein cholesterol, LDL-C: Low-density lipoprotein cholesterol, AST: Aspartate aminotransferase, ALT: Alanine aminotransferase, GHb: glycosylated hemoglobin, MNCV: motor nerve conduction velocity, Amp.: amplitude, CMAP: compound muscle action potential, SNCV: sensory nerve conduction velocity, SNAP: sensory nerve action potential, F-min: F-wave minimum latency, SBP: systolic blood pressure, DBP: diastolic blood pressure, CV_R-R_: coefficient of variation of R-R intervals, baPWV: brachial-ankle pulse wave velocity, ERG: electroretinogram, Td-s: troland-seconds.

### Correlation of the DR score with clinical parameters

The DR score demonstrated noteworthy correlations with various clinical parameters, including the duration of diabetes, eGFR, urinary albumin-to-creatinine ratio, sural SNCV, tibial MNCV, tibial CMAP amplitude, the severity of DPN as classified by the BDC (stages 0–4), deep breathing CV_R-R_, as well as the implicit time and amplitude in ERG (Table 2).

**Table 2:** Correlation between the DR score and clinical parameters.

	Correlation coefficient
Duration of diabetes (years)	0.242^*^
Age (year)	−0.102
BMI	−0.004
SBP (mmHg)	0.005
DBP (mmHg)	−0.005
Random blood glucose (mg/dl)	0.074
GHb (%)	−0.031
Creatinine (mg/dl)	0.178
eGFR (ml/min/1.73 m^2^)	−0.274^*^
uACR (mg/g)	0.410^**^
uCPR (µg/day)	−0.149
Total cholesterol (mg/dl)	0.173
HDL (mg/dl)	0.040
LDL (mg/dl)	−0.002
Triglyceride (mg/dl)	0.142
IMT mean (mm)	−0.003
ABI	−0.038
baPWV	0.095
Tibial nerve	
NCV (m/s)	−0.437^**^
Amplitude (mV)	−0.321^**^
F wave latency (ms)	0.278^**^
Sural nerve	
NCV (m/s)	−0.391^**^
Amplitude (µV)	−0.174
CV_R-R_, resting (%)	−0.196
CV_R-R_, deep breathing (%)	−0.305^**^
Severity of DPN in BDC	0.281^**^
ERG implicit time, 16 Td-s (ms)	0.822^**^
ERG amplitude, 16 Td-s (µV)	−0.566^**^
ERG implicit time, 32 Td-s (ms)	0.861^**^
ERG amplitude, 32 Td-s (µV)	−0.604^**^

SBP: systolic blood pressure, DBP: diastolic blood pressure, GHb: glycosylated hemoglobin, eGFR: estimated glomerular filtration rate, uACR: urine albumin-to-creatinine ratio, HDL-C: High-density lipoprotein cholesterol, LDL-C: Low-density lipoprotein, IMT: intima-media thickness, ABI: Ankle-brachial index, baPWV: brachial-ankle pulse wave velocity, CV_R-R_: coefficient of variation of R-R intervals, DPN: diabetic polyneuropathy, BDC: Baba’s Differentiation Classification, ERG: electroretinography, Td-s: troland-seconds, *: p < 0.05, **: p < 0.01.

### Estimation of the severity of DPN using the DR score

To evaluate the utility of the DR score in diagnosing DPN, we examined its relationship with the stages of DPN classified by the BDC. A multiple regression analysis was performed with BDC stage numbers as the dependent variable, and age, sex, and the DR score as independent variables. The analysis revealed that both the DR score and age were significant predictors of DPN severity, with p-values of 0.004 and 0.006, respectively, indicating that these factors collectively contributed to the variance in DPN stages. The resulting model for estimating the severity of DPN (eBDC) was expressed by the equation: eBDC = −1.112 + 0.057 × DR score + 0.018 × age (years), with an r-value of 0.406 (Table 3).

**Table 3:** Model summary of the multiple regression analysis for the severity of DPN.

Model summary					
Multiple R				0.406	
R^2^				0.165	
Adjusted R^2^				0.143	
Standard error				0.860	
Coefficients: variables included in the equation					
	Unstandardized coefficients		Standardized coefficients		
	B	S.E.	β	*t*	Significance
Constant	−1.112	0.589		−1.886	0.063
DR score	0.057	0.019	0.314	2.997	0.004
Age	0.018	0.007	0.295	2.814	0.006
Variables not in the equation					
	Beta In	*t*	Significance	Partial correlation	Tolerance
Sex	−0.019	−0.178	0.859	−0.020	0.959

S.E.: standard error, Beta In: Standardized regression coefficient at entry, Tolerance: Tolerance for collinearity statistics.

**Fig 1:**
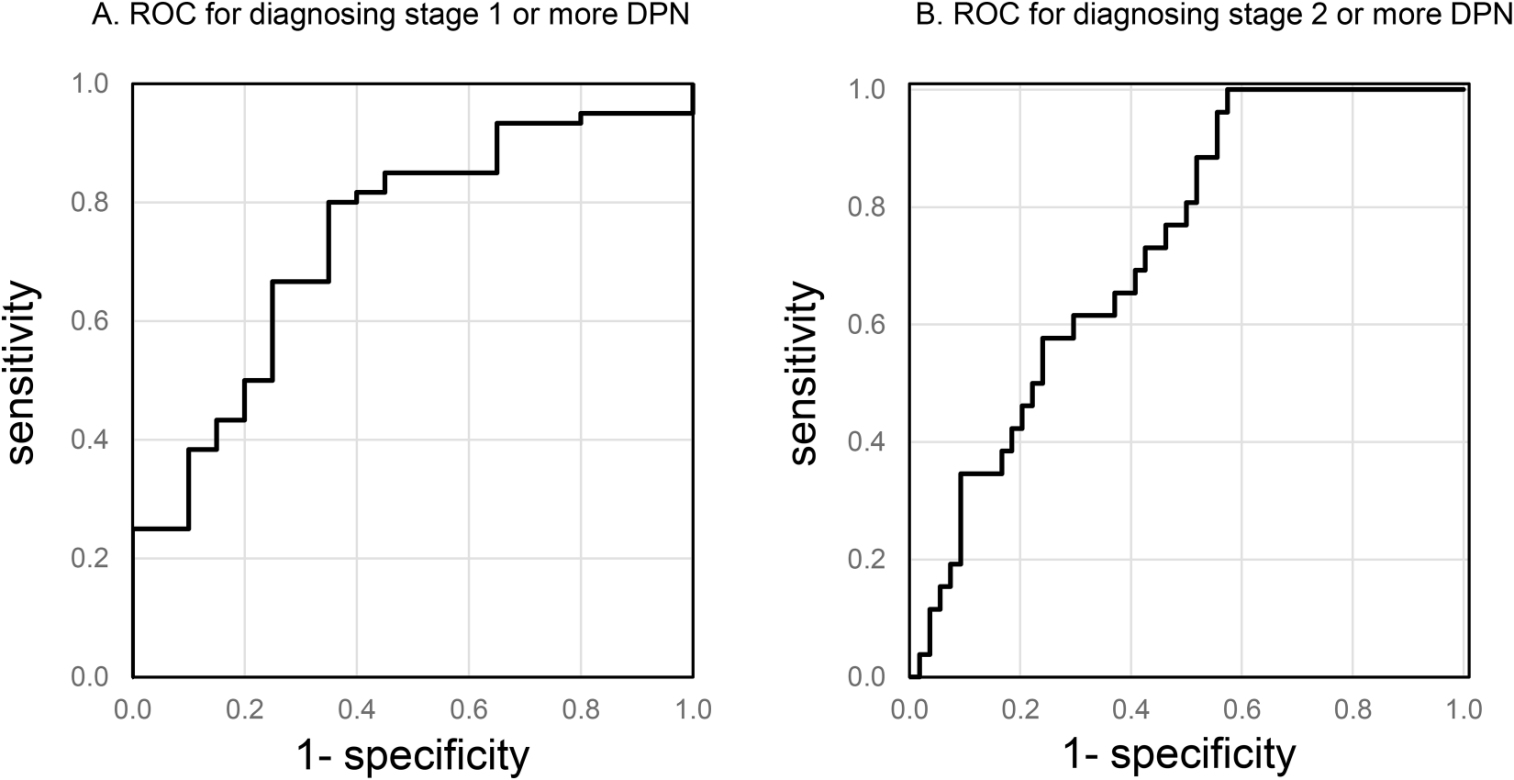
Receiver operating characteristic (ROC) curve. ROC curves validate the diagnostic potential of the equation to predict the estimated severity of diabetic polyneuropathy. **A.** ROC curve to predict stage 1 or higher of diabetic polyneuropathy in Baba’s differentiation classification. **B.** ROC curve to predict stage 2 or higher of diabetic polyneuropathy in Baba’s differentiation classification.

The ROC analysis was employed to ascertain the most appropriate threshold on the eBDC for the classification of individuals as possessing stage 1 or higher of DPN. The eBDC demonstrated a moderate discriminative ability, as evidenced by an AUROC of 0.738 (Fig 1A). The threshold that yielded the highest accuracy was identified as 0.942 on the eBDC scale, corresponding to a sensitivity of 80.0% and a specificity of 65.0%, with a positive predictive value of 87.3%, a negative predictive value of 52.0%, a positive likelihood ratio of 2.29, and a negative likelihood ratio of 0.31. At this threshold (0.942), age, DR score, and eBDC were all significantly higher in participants classified as having BDC stage ≥1 compared with those at stage 0 (S1 Table). S2 Table summarizes the cross-tabulation of observed BDC stages against eBDC-based classifications (≥0.942 or **<**0.942).

Moreover, ROC analysis was conducted using the eBDC to classify participants as having stage 2 or higher of DPN, with a view to elucidating the practical utility of the DR score. Herein, the eBDC exhibited a comparable discriminative capacity, reflected in an AUROC of 0.729 (Fig 1B). The threshold for optimal accuracy was determined to be 0.917, yielding a sensitivity of 61.5% and a specificity of 70.4%, with the positive predictive value, negative predictive value, positive likelihood ratio, and negative likelihood ratio being 50.0%, 79.2%, 2.08, and 0.55, respectively.

## Discussion

The findings of this study illuminate the potential utility of the DR score, as derived from the RETeval™ system, in the early detection and staging of DPN. Our investigation underscores the pertinence of exploring alternative diagnostic avenues that may alleviate the practical constraints associated with NCS.

The DR score emerges from our analysis as a meaningful predictor of DPN severity. The correlation between the DR score and various clinical parameters traditionally associated with DPN—including nerve conduction velocities, amplitudes, and CV_R-R_—bolsters the argument for its broader diagnostic applicability. The multiple regression analysis further corroborates this, revealing the DR score as a significant determinant of DPN stage when adjusted for age and sex.

Our study’s ROC analysis demonstrates a moderate discriminative ability of the eBDC, particularly in distinguishing between stage 0 and 1 of DPN. Although the area under the ROC curve values indicate that the DR score’s predictive power is not absolute, the sensitivity and specificity achieved at the identified thresholds are promising. This suggests that while the DR score alone may not supplant NCS, it could serve as an adjunctive tool, providing clinicians with a preliminary indication of DPN severity that could guide further diagnostic investigation.

A noteworthy finding in our study is the DR score’s ability to identify BDC stage 1 or more severe of DPN, which is particularly challenging to diagnose. BDC stage 1 is characterized by early nerve conduction deficits and typically lacks patient-reported symptoms, as well as clinical signs such as decreased or absent Achilles tendon reflexes and diminished vibration perception. These features make diagnosing BDC stage 1 without NCS exceedingly difficult. Although we have previously reported the utility of the DPNCheck device in predicting BDC stage 2 or higher [14], it proved inadequate for predicting BDC stage 1. The DR score, therefore, stands out as a rare and valuable tool in this regard.

One must consider the significance of these findings in the context of existing diagnostic frameworks. The Toronto Consensus has long emphasized the necessity of NCS for a definitive DPN diagnosis, yet the financial and logistical demands of such studies have limited their widespread application. The RETeval™ system, by contrast, offers a less invasive and more rapid assessment, which, when paired with the DR score, may offer a pragmatic alternative, particularly in settings where resources are constrained.

When using the eBDC cut-off values to calculate the DR score cut-off for a hypothetical 60-year-old patient, the DR score cut-off for diagnosing BDC stage 1 or higher is found to be 17.1, and for diagnosing BDC stage 2 or higher, it is 21.4. Given that the DR score cut-off for predicting DR has been reported as 22 [20], it is noteworthy that the DR score cut-off for diagnosing BDC stage 1 or higher is considerably lower. This observation is consistent with the general concept that diabetic microvascular complications manifest sequentially in the order of DPN, DR, and diabetic nephropathy.

However, this study is not without limitations. The retrospective nature of the analysis and the relatively small sample size may introduce bias and limit the generalizability of our findings. Furthermore, the proprietary nature of the DR score algorithm poses a challenge to full transparency in the evaluation of its diagnostic utility. Future investigations should aim to validate these findings in larger, prospective cohorts and explore the underlying mechanisms that link the DR score’s components to DPN.

Importantly, the RETeval™ system’s portability and user-friendly design may facilitate its application in primary care settings. This could enable broader early screening for DPN outside specialized centers, potentially contributing to timely interventions and improved patient outcomes.

In conclusion, while the DR score, as integrated within the RETeval™ system, shows promise as a tool for the early detection of DPN, further research is warranted to fully elucidate its role within the diagnostic landscape. Should subsequent studies corroborate our findings, the adoption of this method could represent a meaningful advancement in the management of diabetic patients at risk for neuropathy, ultimately contributing to improved clinical outcomes.

## Supporting information

S1 Table Comparison of age, DR score, and eBDC values stratified by the optimal eBDC cutoff (0.9423) for predicting BDC stage ≥1. (DOCX)

S2 Table Cross-tabulation of BDC stages (0–4) and the number of patients with eBDC values above or below the cutoff of 0.9423. (DOCX)
