# Supplementary material for: The DR score in RETeval™ electroretinogram system facilitates expeditious and uncomplicated early detection and assessment of diabetic polyneuropathy in clinical practice
Source: PLoS One. 2025 Nov 13;20(11):e0336117. doi: 10.1371/journal.pone.0336117 (PMC12614585; doi:10.1371/journal.pone.0336117)
Supplement: S1 Table — (DOCX) [file pone.0336117.s001.docx]

**Supplementary Table 1.** Comparison of age, DR score, and eBDC values stratified by the optimal eBDC cutoff (0.9423) for predicting BDC stage ≥1.

|  | eBDC < 0.9423 | eBDC ≥ 0.9423 | *p* value |
| --- | --- | --- | --- |
| Number | 25 | 57 |  |
| Age | 48.9±10.5 | 67.4±12.9 | <0.001 |
| eBDC | 0.737±0.144 | 1.310±0.304 | <0.001 |
| DR score, ERG | 17.0±2.4 | 21.2±5.5 | <0.001 |

ERG: electroretinogram, eBDC: estimating the severity of DPN
