# Supplementary material for: The DR score in RETeval™ electroretinogram system facilitates expeditious and uncomplicated early detection and assessment of diabetic polyneuropathy in clinical practice
Source: PLoS One. 2025 Nov 13;20(11):e0336117. doi: 10.1371/journal.pone.0336117 (PMC12614585; doi:10.1371/journal.pone.0336117)
Supplement: S2 Table — (DOCX) [file pone.0336117.s002.docx]

**Supplementary Table 2.**
Cross-tabulation of BDC stages (0–4) and the number of patients with eBDC values above or below the cutoff of 0.9423.

|  | eBDC < 0.9423 | eBDC ≥ 0.9423 |
| --- | --- | --- |
| BDC stage 0 | 13 (16.3%) | 7 (8.8%) |
| BDC stage 1 | 11 (13.8%) | 23 (28.7%) |
| BDC stage 2 | 1 (1.3%) | 22 (27.5%) |
| BDC stage 3 | 0 (0.0%) | 0 (0.0%) |
| BDC stage 4 | 0 (0.0%) | 3 (3.8%) |

BDC: Baba’s Differentiation Classification, eBDC: estimating the severity of DPN
